# Purification of NAD^+^ glycohydrolase from human serum

**DOI:** 10.3892/ol.2013.1335

**Published:** 2013-05-08

**Authors:** ÖZLEM COŞKUN, RÜSTEM NURTEN

**Affiliations:** 1 Department of Biophysics, Onsekiz Mart University Faculty of Medicine, Çanakkale 17100;; 2 Department of Biophysics, Istanbul Faculty of Medicine, University of Istanbul, Çapa, Istanbul 34104, Turkey

**Keywords:** ADP-ribose, affinity chromatography, isoelectric focusing, NAD glycohydrolase, soluble CD38

## Abstract

In the present study, NAD^+^ glycohydrolase was purified from serum samples collected from healthy individuals using ammonium sulfate fractionation, Affi-Gel blue (Cibacron Blue F3GA) affinity chromatography, Sephadex G-100 column chromatography and isoelectric focusing. The final step was followed by a second Sephadex G-100 column chromatography assay in order to remove the ampholytes from the isoelectric focusing step. In terms of enhancement of specific activity, the NAD^+^ glycohydrolase protein was purified ∼480-fold, with a yield of 1% compared with the initial serum fraction. The purified fraction appeared to be homogeneous, with a molecular weight of 39 kDa, as revealed by sodium dodecyl sulfate-polyacrylamide gel electrophoresis (SDS-PAGE) analysis, and also corresponded to the soluble (monomeric) form of surface antigen CD38.

## Introduction

NAD^+^ glycohydrolase (NADase; EC 3.2.2.5) is an enzyme that catalyzes the hydrolysis of NAD^+^ to nicotinamide and ADP-ribose. In eukaryotes, particularly mammals, NAD^+^ glycohydrolase activity is largely attributed to surface antigen CD38, a multifaceted protein detected primarily in the activation stages of lymphocytes (1,2). CD38 is exposed as a transmembrane protein on the surface of hematopoietic cells and several other cell types, and is involved in heterotypic interactions with CD31 on endothelial cells, as well as acting as a receptor that modulates signal transmission. Finally, CD38 is an ectoenzyme with its catalytic site residing on the outer surface of the cell membrane (3). As such, it exhibits three catalytic activities, those of NAD^+^ glycohydrolase, ADP-ribosyl cyclase and cyclic ADP-ribosyl hydrolase. Cyclic ADP-ribose, the product of CD38 ADP-ribosyl cyclase activity, has received considerable interest as an inositol 1,4,5-triphosphate (IP-3)-independent Ca^2+^-mobilizer. Moreover, CD38 expression has gained importance as a prognostic marker in chronic lymphocytic leukemia (CLL) (4,5), HIV infection (6) and cancer (7,8).

A soluble form of CD38 with a molecular weight of 38 kDa also exists. The form was first identified in the supernatants of a mixed lymphocyte culture and CD38^+^ leukemic cell lines (9). The soluble form appears to correspond to the extracellular portion of CD38 and exists partially in dimeric form (10). The presence of elevated levels of a soluble form of CD38 has also been shown in serum samples from cancer patients (11). Soluble CD38 was reported to be decreased in rheumatoid arthritis and systemic lupus erythematosus patients (12), but increased in carriers of viral hepatitis G markers (13). A decrease in the serum levels of the dimeric form of soluble CD38 was observed in burned patients (14). By contrast, CD38 has been implicated to be involved in the prolongation of the lifespan of memory B-cell responses (15). Such data suggests a possible association between CD38 and various physiological and pathological events. The levels of soluble CD38 appear to change depending on the underlying disorder. In view of such variable data, the present study attempted to purify soluble CD38, as the availability of purified protein would enable the identification of its molecular and cellular interactions and eventually the role(s) that CD38 may have in various pathogenic networks.

## Materials and methods

### 

#### Materials

[Carbonyl-^14^C]NAD^+^, with a specific activity of 53 mCi/mmol, was purchased from Amersham Life Sciences (Piscataway, NJ, USA). Sephadex G-100, Affi-Gel blue (Cibacron Blue F3GA), monoclonal anti-human CD38 clone HI157, NAD^+^ glycohydrolase (from a Pig’s brain), Ampholine carrier ampholytes and all chemicals of analytical grade were obtained from Sigma-Aldrich (St. Louis, MO, USA). The AG1-X4 anion exchange resin was a product of Bio-Rad Laboratories (Hercules, CA, USA).

Serum samples were obtained from young and apparently healthy individuals.

### Purification of NAD^+^ glycohydrolase from serum

#### Steps

All procedures were performed at 4°C. The serum samples were fractionated using successive steps of ammonium sulfate precipitation, affinity chromatography (Affi-Gel blue), gel filtration chromatography (Sephadex G-100) and isoelectric focusing.

*Ammonium sulfate fractionation.* Well-powdered solid ammonium sulfate was added slowly to the serum samples and was continuously stirred until saturation levels of 35 and 65% were attained. Once each level of saturation had been reached, the stirring was continued for a further 30 min. Thereafter, the samples were centrifuged for 20 min at 10,000 x g and the collected precipitated proteins were dissolved in and dialyzed exhaustively against 10 mM potassium phosphate (pH 7.4). The supernatant obtained following the centrifugation of the precipitated proteins at 65% saturation was also dialyzed against the same buffer.

*Affinity (Cibacron Blue F3GA) chromatography.* Affinity chromatography was performed with an Affi-Gel blue column as follows (16). The column (1.5×30 cm) was equilibrated with buffer A [10 mM potassium phosphate, pH 7.4, containing 0.5% 3-[(3-cholamidopropyl)dimethylammonio]-2-hydroxy-1-propane sulphonate (CHAPS)]. Subsequent to adding the sample, the column was washed with buffer A and enzymatic activity was eluted with the same buffer containing 1.5 M NaCl.

*Gel filtration chromatography.* Fractionation was performed at 4°C on a Sephadex G-100 Superfine gel. The column (1×50 cm) was equilibrated with 50 mM Tris-HCl, pH 7.4 and 100 mM KCl and calibrated using bovine serum albumin (M_r_, 66 kDa), ovalbumin (M_r_, 45 kDa) and carbonic anhydrase (M_r_, 29 kDa) as molecular weight markers. The column was eluted with the same buffer at a flow rate of 0.05 ml/min. The amount of protein in the 1-ml column fractions was monitored by absorption measurements at 280 nm.

*Isoelectric focusing.* Following the gel filtration chromatography, the fractions with molecular weights of 35-40 kDa and the NAD^+^ glycohydrolase activity were collected, dialyzed against 10 mM Tris-HCl (pH 7.4) and subjected to isoelectric focusing with an LKB 8100-1 column, according to the manufacturer’s instructions. Briefly, half of the dialyzed protein sample was mixed with 1.8 ml carrier ampholytes (pH range, 3.5–10.0) in a total volume of 60 ml. This solution also contained 28 g sucrose (dense solution). The other half of the protein sample was mixed with 0.6 ml carrier ampholytes (pH range, 3.5–10.0), again in a total volume of 60 ml (light solution). The two solutions were mixed in a series of reaction tubes so that a density gradient was formed and, thereafter, layered in a stepwise manner in the column over the anode solution (0.2 ml phosphoric acid, 12 g sucrose and 14 ml dH_2_O). Finally, the cathode solution (0.2 ml ethylenediamine and 10 ml dH_2_O) was added to the top of the gradient. The isoelectric focusing was performed for 24 h at 4°C and the operational power level was maintained at <W. Upon completion, the column content was harvested in 1-ml fractions and their A_280_, pH-values and enzymatic activities were determined.

*Gel filtration chromatography.* Following isoelectric focusing, the fractions with NAD^+^ glycohydrolase activity were once again subjected to Sephadex G-100 chromatography, as described previously, in order to remove the ampholytes.

#### NAD^+^ glycohydrolase activity assay

NAD^+^ glycohydrolase activity was determined by separating [carbonyl-^14^C]nicotinamide from [carbonyl-^14^C]NAD^+^ with a BioRad AG-1X4 anion exchange resin (17). The reaction mixture (20 *μ*l), containing 12 *μ*l serum fractions, 7 *μ*l mix (10 mM NaCl, 500 *μ*M ZnCl_2_, 50 *μ*M CaCl_2_ and 20 mM Tris-HCl, pH 9.0) and 10 *μ*M [carbonyl-^14^C]NAD^+^, was incubated for 30 min at 37°C. The samples were then added to the BioRad AG-1X4 column and the [carbonyl-^14^C]nicotinamide that was released due to NAD^+^ glycohydrolase action was eluted with dH2O. The [carbonyl-^14^C]NAD^+^ that was retained on the column was subsequently eluted with 0.5 M NaCl. The radioactivity that was eluted with dH_2_O and 0.5 M NaCl was determined in Bray’s solution using a liquid scintillation counter (Packard Tri-Carb 1000TR, Meriden, CT, USA). The specific activity of the NAD^+^ glycohydrolase activity was defined as the nmol of [carbonyl-^14^C]nicotinamide released under the aforementioned reaction conditions per mg of protein.

#### Sodium dodecyl sulfate-polyacrylamide gel electrophoresis (SDS-PAGE)

SDS-PAGE was performed as described previously (18). Bovine serum albumin (M_r_, 66 kDa), ovalbumin (M_r_, 45 kDa), carbonic anhydrase (M_r_, 29 kDa) and trypsinogen (M_r_, 24 kDa) were used as molecular weight standards. Aliquots (∼10 *μ*l) of enzyme preparations in sample buffer were heated for 3 min in a boiling water bath prior to being added to the gel. The protein bands in the gel slabs were visualized with silver staining (19).

## Results and Discussion

### Purification of NAD^+^ glycohydrolase

#### Ammonium sulfate precipitation

**Figure 1.**
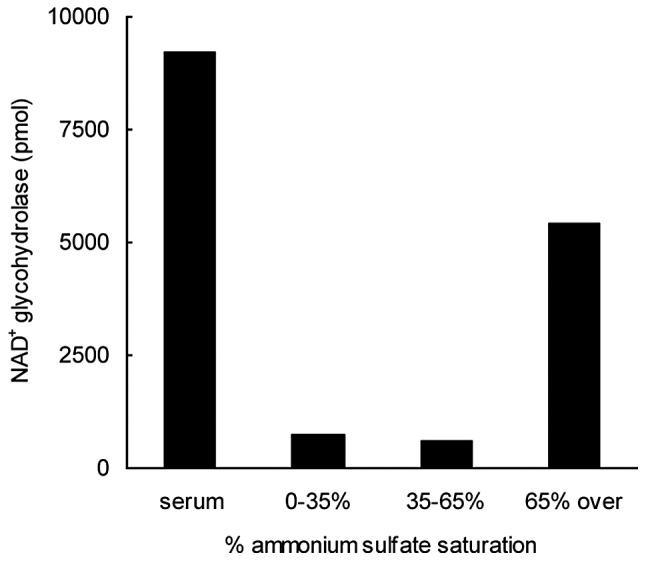
NAD^+^ glycohydrolase activity of serum fractions obtained by ammonium sulfate precipitation. Serum proteins were fractionated by precipitation with ammonium sulfate at saturations of 35 and 65%. The precipitated proteins collected by centrifugation, as well as the supernatant fraction obtained following centrifugation of the proteins precipitated at 65% ammonium sulfate saturation, were dialyzed against 10 mM potassium phosphate, pH 7.4, and then assayed for NAD^+^ glycohydrolase activity. Bars represent the total activity present in each fraction. In this pilot fractionation performed with 20 ml serum, the yield was considerably higher than that attained when larger serum volumes were processed (see Table I for comparison).

Serum (60 ml) was subjected to fractionation with ammonium sulfate at concentrations that were increased in a stepwise manner, first to 35 and then to 65% saturation. Following each saturation step, the precipitated proteins were collected by centrifugation, dissolved in and dialyzed against 10 mM potassium phosphate, pH 7.4, and assayed for NAD^+^ glycohydrolase activity (Fig. 1). The supernatant fraction obtained following the centrifugation of the proteins precipitated at 65% ammonium sulfate saturation was also subjected to dialysis and a subsequent activity test. NAD^+^ glycohydrolase activity was observed almost exclusively in the 65% supernatant. This step resulted in the removal of a significant portion of the serum proteins and in an ∼20-fold enrichment of the enzymatic activity, with a yield of 32% (Table I).

**Figure 2.**
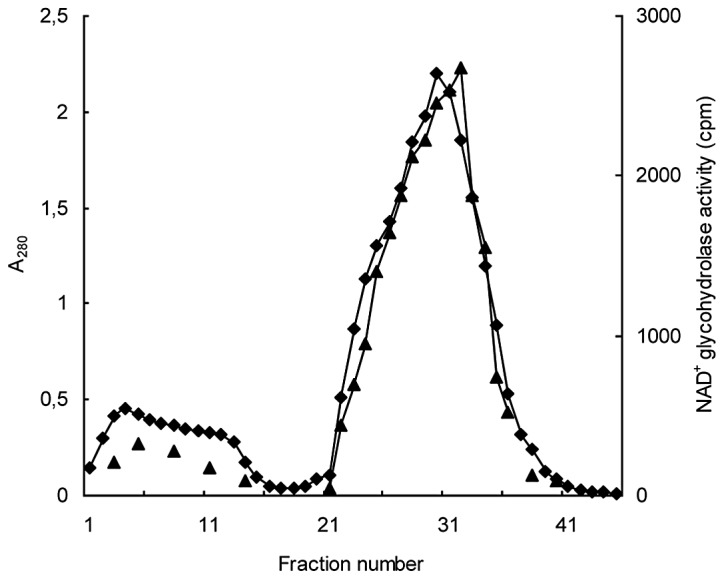
Attempt to purify NAD^+^ glycohydrolase by Affi-Gel blue affinity chromatography. The supernatant fraction obtained by ammonium sulphate precipitation at 65% saturation was subjected to Affi-Gel blue affinity chromatography and the 1-ml fractions collected were assayed for NAD^+^ glycohydrolase activity. ▴-▴, NAD^+^ glycohydrolase activity; ♦-♦, A_280_.

*Affi-Gel blue affinity chromatography.* The 65% supernatant fraction was then injected into the Affi-Gel blue column from which proteins were successively eluted with buffer A and buffer A plus 1.5 M NaCl. A stepwise elution approach at lower NaCl concentrations failed to isolate the activity in a well-defined concentration range. Instead, the activity was observed to be spread over all the attempted concentrations. Moreover, the 1.5 M NaCl-eluate appeared to contain the bulk of the protein that was applied to the column, aside from the NAD^+^ glycohydrolase activity. Thus, this step did not result in a meaningful enrichment of the enzymatic activity (Fig. 2).

**Figure 3.**
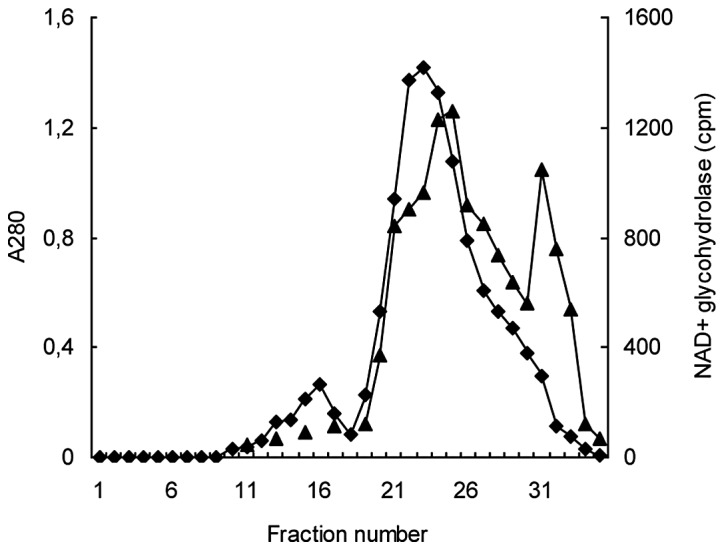
Fractionation of serum NAD^+^ glycohydrolase activity by Sephadex G-100 column chromatography. Following Affi-Gel blue affinity chromatography, the fractions with NAD^+^ glycohydrolase activity were pooled and a 5 ml aliquot was applied to the Sephadex G-100 column. Fractions of 1 ml were collected and their NAD^+^ glycohydrolase activities were determined. ▴-▴, NAD^+^ glycohydrolase activity; ♦-♦, A_280_.

*Gel filtration chromatography (Sephadex G-100).* Following Affi-Gel blue affinity chromatography, the 1.5 M NaCl-eluate was subjected to gel filtration using a Sephadex G-100 column. This step resulted in the separation of the NAD^+^ glycohydrolase activity into peaks, with molecular weight ranges of 70–75 kDa and 35–40 kDa. The major peak with the higher molecular weight, which comprised more than two-thirds of the total NAD^+^ glycohydrolase activity, also overlapped with the main A_280_ peak. The minor, sharp activity peak directly followed the descending section of the A_280_ peak. Having been separated from the bulk of the protein, this peak (35–40 kDa) revealed a relatively high specific activity and appeared to be the most suitable for the further purification steps (Fig. 3).

**Figure 4.**
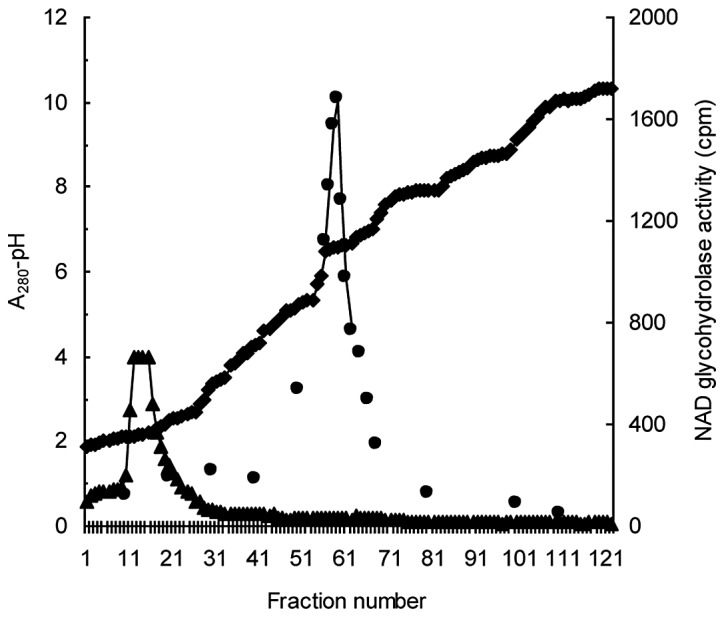
Isoelectric focusing of serum NAD^+^ glycohydrolase. Following Sephadex G-100 column chromatography, the fractions corresponding to the minor peak of NAD^+^ glycohydrolase activity within the molecular weight range of 35–40 kDa were combined, dialyzed against 10 mM Tris-HCl, pH 7.4 and mixed with ampholytes, then subjected to isoelectric focusing. The pH, A_280_ and NAD^+^ glycohydrolase activity of the fractions were determined at the end of the process. ♦-♦, pH; ▴-▴, A_280_; •-•, NAD^+^ glycohydrolase activity.

*Isoelectric focusing.* The fractions from the Sephadex G-100 column chromatography that corresponded to the minor activity peak within the molecular weight range of 35–40 kDa were pooled and subjected to isoelectric focusing. At the end of the isoelectric focusing, NAD^+^ glycohydrolase activity was observed in the pH range of 6.4–6.6 (Fig. 4). The active fractions had low A_280_ values, whereas the main A_280_ peak was located at the bottom of the column and pH-gradient. The active fractions were collected and applied once more to the Sephadex G-100 column in order to remove the ampholytes.

#### Assessment of purification

**Figure 5.**
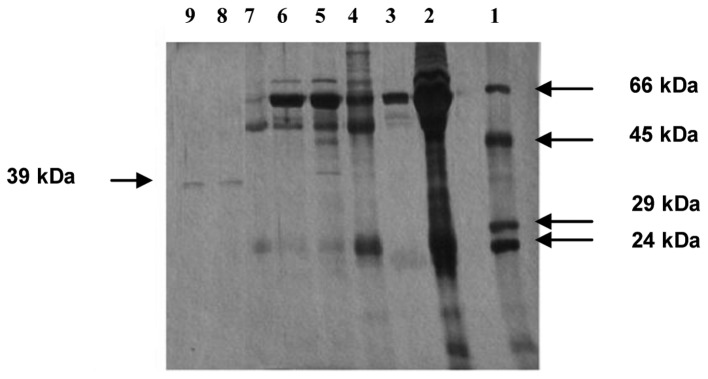
SDS-PAGE analysis of serum NAD^+^ glycohydrolase fractions following successive purification steps. Bovine serum albumin (M_r_, 66 kDa), ovalbumin (M_r_, 45 kDa), carbonic anhydrase (M_r_, 29 kDa) and trypsinogen (M_r_, 24 kDa) were used as molecular weight standards. The gel lanes, in order from 1–9, correspond to protein molecular weight standards, unfractionated serum proteins, supernatant of 65% ammonium sulfate saturation, eluate from Affi-Gel blue chromatography (fractions 24–34), eluate from Sephadex G-100 chromatography (fractions 20–23), eluate from Sephadex G-100 chromatography (fractions 30–33), eluate from isoelectric focusing (fractions 58–60), eluate from second Sephadex G-100 chromatography (fraction 31), eluate from second Sephadex G-100 chromatography (fraction 32). SDS-PAGE, sodium dodecyl sulfate-polyacrylamide gel electrophoresis.

A SDS-PAGE analysis of the active fraction obtained at the end of the purification procedure revealed the presence of a single protein band that corresponded to a molecular weight of ∼39 kDa (Fig. 5). This value appears to be in line with the molecular weight of the monomeric form of soluble CD38 that has been reported previously (9,10).

We suggest that the soluble monomer of CD38 was purified from the human serum in the present study since the molecular weight of the purified protein matched that reported for soluble monomeric CD38 (9,10). It is notable that the second gel filtration step performed on the Sephadex G-100 column with the aim of depleting the ampholytes, actually led to a final, clear enrichment of this monomeric form of soluble NAD^+^ glycohydrolase. The findings of the SDS-PAGE analysis are also in line with the considerable increase in specific activity obtained subsequent to the second gel filtration step (Table I). It appears that a more efficient fractionation by gel filtration was possible only following the removal of the serum albumin load by ammonium sulfate fractionation and isoelectric focusing.

As indicated in Table I, the NAD^+^ glycohydrolase was purified by 483-fold compared with the starting serum fraction, although the yield of the purification was only 1%. We hypothesize that there are two main reasons for this modest yield. Firstly, the Affi-Gel blue step resulted in the loss of a significant part of the activity with practically no gain in specific activity. Accordingly, a ‘cost-benefit analysis’ does not justify the inclusion of this step in the present purification procedure, at least not at such an early stage. Affi-Gel blue chromatography has previously been used successfully in the purification of proteins with dinucleotide fold (20) and NAD^+^ glycohydrolase from calf spleens and thyroid glands (16,21). However, the present study demonstrated that Affi-Gel blue chromatography was of little use in the purification of serum NAD^+^ glycohydrolase, since serum albumin also exhibits a marked affinity for Cibacron Blue F3GA. The affinity of serum albumin for Cibacron Blue F3GA has resulted in an expansion in the development of chromatographic methods for albumin depletion in the preparation of low abundance proteins for proteomic analysis (22–26). In the present study, a stepwise development of Affi-Gel blue affinity chromatography, with the aim of differentially separating NAD^+^ glycohydrolase from serum albumin, was not successful. Moreover, the co-elution of the major, high molecular weight (possibly dimeric) form of this protein together with serum albumin in gel filtration chromatography dissuaded us from concentrating on its purification. That the purification of this dimeric form was not attempted is thus the second reason accounting for the low yield of the present procedure.

However, a change in the purification protocol, with the isoelectric focusing step directly following ammonium sulfate fractionation, may enable the efficient separation of the two forms of NAD^+^ glycohydrolase from serum albumin. The fairly low isoelectric point (pI) of serum albumin (4.4) (27) provides an opportunity which may be exploited.

In the present attempt to purify NAD^+^ glycohydrolase (soluble CD38) from human serum, the isolation of its monomeric form in an apparently homogeneous state was achieved. However, the abundance of serum albumin and its co-elution in Affi-Gel blue affinity chromatography, as well as gel filtration with Sephadex G-100, prevented the similar purification of the putative dimer of NAD^+^ glycohydrolase. This result demonstrates the difficulties that are likely to be encountered in the purification of low abundance proteins from serum when a differential depletion of serum albumin is not possible.

The information gained in this first attempt provides valuable information for designing future approaches that should take into account the similar chromatographic behavior of the NAD^+^ glycohydrolase dimer and serum albumin. Therefore, we propose that purification protocols that initially deplete serum albumin, particularly by making use of its low pI-value, are likely to have an improved prospect for the purification of the monomeric and dimeric forms of NAD^+^ glycohydrolase. At present, research is in progress to aid in the implementation of this strategy.

## Figures and Tables

**Table I.** Purification of NAD^+^ glycohydrolase from human serum.

Fraction	Total protein (mg)	Volume (ml)	Total activity (nmol)	Specific activity (nmol/mg)	Purification (fold)	Yield (%)
Serum	4920	60	285	0.058	1	100
Ammonium sulfate	80	34	92	1.15	19.8	32.3
Affi-Gel Blue	17	10	21	1.24	21.4	7.4
Sephadex G-100 (1)	0.94	4	3.3	3.5	60.3	1.15
Isoelectric focusing	0.49	3	3.5	7.1	122.4	1.23
Sephadex G-100 (2)	0.1	3	2.8	28	483	0.97
